# IgG4-related disease: a systematic review of this unrecognized disease in pediatrics

**DOI:** 10.1186/s12969-016-0079-3

**Published:** 2016-03-25

**Authors:** Faiz Karim, Jan Loeffen, Wichor Bramer, Lauren Westenberg, Rob Verdijk, Martin van Hagen, Jan van Laar

**Affiliations:** Departments of Internal Medicine and Immunology Erasmus MC, ’s-Gravendijkwal 230, 3015 CE Rotterdam, The Netherlands; Department of Pediatrics Oncology, Erasmus MC-Sophia Children’s hospital, Rotterdam, The Netherlands; Medical library, Erasmus MC, Rotterdam, The Netherlands; Department of Pathology, Erasmus MC, Rotterdam, The Netherlands

## Abstract

**Background:**

Immunoglobulin G4-related disease (IgG4-RD) is a systemic fibro-inflammatory condition with an unclear pathophysiological mechanism affecting different parts of the body. If untreated, the disease can lead to fibrosis and irreversible organ damage. IgG4-RD mostly has been described in adults, hence it is generally unknown among pediatricians. This systematic search of the literature provides an overview of all reports published on IgG4-RD in children in order to create awareness of IgG4-RD in pediatrics and to emphasize the broad clinical presentation of this disease.

**Methods:**

A systematic literature search of Embase, Medline, Web-of-Science, PubMed publisher, Cochrane and Google Scholar was performed for case reports on IgG4-RD in children.

**Results:**

Of total 740 articles identified by the search, 22 case reports including 25 cases of IgG4-RD in children were found. The median age of the children was 13 years, of which 64 % were girls. IgG4-related orbital disease (44 %) and autoimmune pancreatitis type 1/IgG4-related pancreatitis (12 %) predominantly occurred. Less frequently, other manifestations as pulmonary manifestation, cholangitis and lymphadenopathy were also found. Almost all cases were histologically proven. Prednisone was the first choice of treatment leading to favorable clinical response in 83 % of the cases. Maintenance therapy with steroid sparing agents was required in 43 % of the cases needing therapy. Rituximab was successful in all 4 cases, whereas, the disease modifying rheumatic drugs (DMARDs) mycophenolate mofetil, azathioprine and methotrexate were effective in almost 50 % of the cases.

**Conclusion:**

IgG4-RD in children is a generally unknown disease among pediatricians, but several pediatric cases have been described. Prednisone is the first choice of treatment leading to disease remission in the majority of the cases. DMARDs and rituximab are alternative effective steroid sparing agents with more positive evidence for the latter.

## Background

IgG4-RD is a systemic fibro-inflammatory disease affecting different parts of the body [1]. The disease is characterized by tumour-like infiltrations of IgG4 positive plasma cells in the tissues, mostly with fibrotic abnormalities and often elevated serum IgG4 levels [1]. The underlying pathophysiological mechanism of IgG4-RD is still unclear, but when untreated, the disease can lead to irreversible organ damage because of the fibrosis. Early recognition and therapy is therefore critical [2, 3]. In recent time there has been a lot of attention to IgG4-RD in adult care leading to evolving knowledge about pathogenesis, diagnosis and treatment of this disease. However, further studies are required to provide more insight into this disease, in particular, the underlying pathogenesis has yet to be clarified. The average age at which IgG4-RD can occur, is estimated to be older than 50 years [1, 4]. Although case reports are available on IgG4-RD in children [5, 6], no pediatric studies or reviews about this disease have been published yet. Knowledge and awareness of this disease is essential to prevent missing the diagnosis and subsequent delay of treatment, especially in children.

We performed a systematic literature search in order to make an overview of all the case reports that have been published regarding IgG4-RD in children. The main purpose of this study was to create awareness of IgG4-RD in pediatrics and to emphasize the broad clinical presentation of this disease. Furthermore, with the current knowledge about the disease we wanted to provide an overview on epidemiology, pathogenesis and treatment of this disease for the pediatricians.

## Methods

A systematic literature search was conducted to provide an overview of all case reports and (if available) case series regarding IgG4-RD in pediatrics. The study was performed and reported in accordance with the PRISMA statement for systematic reviews.

### Data source, study selection and data extraction

Relevant articles on IgG4-RD in children were retrieved from Embase.com, Medline (Ovid), Web-of-Science, and the Cochrane Library from inception to last date of inclusion July 16^th^ 2015. Additional references were obtained from PubMed (the subset as supplied by publisher, containing references not yet indexed in Medline) and Google Scholar (the most relevant citations). No filters for date or language were used in the search strategy. See the additional Appendix for the full search strategies for all databases. Two authors reviewed and extracted the data independently.

## Results

**Fig. 1:**
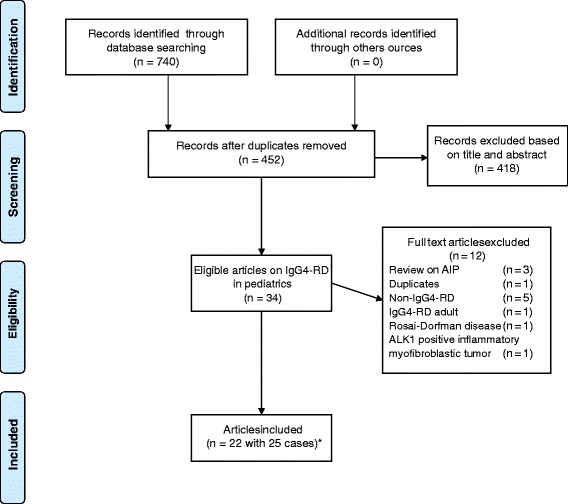
Search strategy and selection of the articles. * Three articles demonstrated each two cases of IgG4-RD in children. Therefore, a total of 25 cases were available for this study

Of a total of 740 articles identified by the search, 34 articles on IgG4-RD in pediatrics were eligible (Fig. 1). After screening, 22 case reports on IgG4-RD in children were identified. Three articles described two pediatric patients leading to a total of 25 cases of IgG4-RD [7–9]. The main outcomes of this study are demonstrated in Table 1.

**Table 1:** Outcomes reported in case reports on IgG4-RD in pediatrics

Reference	Age	Sex	Organ manifestation	Serum IgG4	Therapy	Comments
Miglani 2010 [24]	13y	M	AIP-1 *H+*	El (603 mg/dl)	Pred 20 mg/d	Initially suspected of malignancy. Pred tapered and stopped in 4 months.
Ibrahim 2010 [25]	3y	F	IgG4-R cholangitis *H+*	El (258 mg/dl)	Pred 2 mg/kg/d and Aza 1.5 mg/kg	Relapse after tapering pred and required a low (2 mg/d) maintenance dose of pred and Aza.
Mannion 2010 [14]	13y	F	AIP-1 and IgG4-R fibrosing mediastinitis, renal and hepatic manifestation *H+*	El (73.4 mg/dl)	Pred and MMF	Good results by MMF, pred tapered and stopped successfully.
Zakeri 2011 [20]	17y	M	Riedel’s thyroiditis H + ^a^	NM	Pred 40 mg/d	Pred tapered and stopped in 3 months.
Melo 2012 [28]	11y	M	IgG4-R sialadenitis *H+*	NM	Pred	
Griepentrog 2013 [7]	10y	F	IgG4-ROD *H+*	N (L U)	Lateral orbitotomy	No further treatment was required.
Griepentrog 2013 [7]	14y	F	IgG4-ROD *H+*	N (L U)	Pred, dosage unknown, and MMF	MMF because of relapse after tapering pred, successful.
Kalapesi 2013 [10]	5y	F	IgG4-ROD *H+*	El (1.52 g/l)	Pred 1 mg/kg and MMF (600 mg/m2)	Weaned off pred and maintained on MMF successfully.
Naghibi 2013 [15]	16y	F	IgG4-related colitis, in the past AIP-1 *H+*	El (210 mg/dl)	Adalimumab	Refractory disease to pred 0.5 mg/kg, Aza and infliximab. Adalimumab successful.
Pifferi 2013 [26]	15y	M	IgG4-R pulmonary disease *H+*	El (1090 mg/dl)	Pred 0.6 mg/kg/d	Treatment for 4 weeks.
Sane 2013 [11]	12y	F	IgG4-ROD and nephrotic syndrome *H+*	N (L U)	Methylpred and rituximab	The nephrotic syndrome also resolved. Initial good response to pred 40 mg, but relapse occured.
Pasic 2013 [12]	10y	F	Mikulicz disease/IgG-ROD *H+*	EL 9.02 g/l	NM	
Caso 2013 [16]	17y	M	IgG4-R lymphad and scleritis *H+*	El (4.43 g/l)	Rituximab and pred 10 mg daily	Refractory to MMF, good results with rituximab.
Hasosah 2014 [17]	7y	F	IgG4-R mesenteritis and pericarditis *H+*	El (149 mg/dl)	Pred, aza and colchicine (doses unknown)	Relapsed despite aza, further treatment with 5 mg prednisone as maintenance therapy.
Jariwala 2014 [5]	7y	M	IgG4-ROD *H+*	El (109.3 mg/dl)	Pred 1 mg/kg/d and Aza 2 mg/kg/d	Good clinical results.
Mittal 2014 [6]	14y	M	IgG4-ROD *H+*	El (4.3 g/l)	Pred 0.6 mg/kg/d	Initial improvement, but lost to follow-up.
Notz 2014 [29]	13y	F	IgG4-R dacryoadenitis *H+*	N (23.9 mg/dl)	Pred 40 mg/d for 3 months	
Prabhu 2015 [8]	15y	F	IgG4-ROD and sinonasal disease *H+*	El (579 mg/dl)	Rituximab	Insufficient response to prednisone.
Prabhu 2015 [8]	15 y	F	IgG4-R sinonasal disease *H+*	El (206 mg/dl)	Pred (dosage unknown)	
Batu 2015 [9]	14y	F	IgG4-ROD *H+*	N (7.5 g/l) (0-12.5 g/l)	Pred (dosage unknown)	Pred was tapered and stopped, MTX as maintenance therapy.
Batu 2015 [9]	9y	F	IgG4-ROD *H+*	N (3.7 g/l)	Methylpred and cyclophosphamide	No response to pred, MTX or MMF. Now stable disease.
Corujeira 2015 [18]	22Mo	F	IgG4-R pulmonary disease and IgG4-R lymphad *H+*	El (805 mg/dl)	Pred 2 mg/kg/d	Pred tapered over period of 6 months.
Gillispie 2015 [13]	7y	F	IgG4-ROD, nerve and renal disease *H+*	N (L U)	Pred and rituximab	Refractory to pred, responsive to rituximab.
Nada 2015 [19]	10y	M	IgG4-R hepatic mass and coagulopathy *H+*	El (420 mg/dl)	Pred 2 mg/kg/d	Coagulopathy also resolved after treatment.
Rosen 2015 [27]	17y	M	IgG4-R cholangitis *H+*	El (242 mg/dl)	Pred 30 mg/d	Pred weaned in 3 months.

*Y* year, *IgG4-ROD* IgG4-related orbital disease, *Mo* months, *H+* histology performed, *Mikulicz disease* IgG4-related orbital and submandibular disease, *M* male, *AIP-1* autoimmune pancreatitis type 1, *IgG-R* IgG4-related disease, *F* female, *Pred* prednisone, *Aza* azathioprine, *EL* elevated, *MMF* mycophenolate mofetil, *L U* level unknown, *N* normal, *NM* not measured, *Methylpred* Methylprednisolone, *Lymphad* Lymphadenopathy

^a^Histology without IgG4 staining

### Patients

With this systematic literature review we identified 22 case reports of IgG-RD in children. Identified studies were published over a 5-year span (2010–2015). The case reports included patients aged ranging from 22 months to 17 years of age. The median age of the children in this study was 13 years and 64 % of the children were girls.

### Organ manifestation

**Fig. 2:**
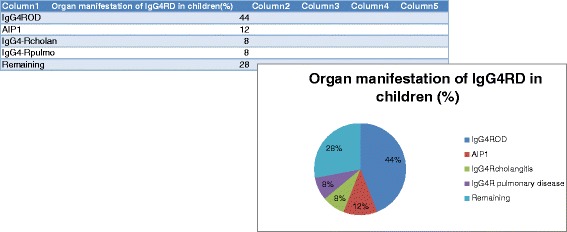
Organ manifestation of IgG4-RD in children. Remaining: Riedel’s thyroiditis/IgG4-related thyroid disease, IgG4-related sialadenitis, IgG4-related mesenteritis, IgG4-related lymphadenopathy, IgG4-related dacryoadenitis, IgG4-related sinonasal disease and IgG4-related hepatic mass

The cases described in this study show a spectrum of different organ manifestations (Fig. 2) of IgG4-RD. However, most of the cases report IgG4-related orbital disease (IgG4-ROD) (44 %) [5–13]. Other manifestations were IgG4-related pancreatitis/autoimmune pancreatitis type 1 (AIP 1) (12 %), IgG4-related cholangitis (8 %), IgG4-related pulmonary disease (8 %), and the remaining cases (28 %) were single cases of Riedel’s thyroiditis/IgG4-related thyroid disease, IgG4-related sialadenitis, IgG4-related mesenteritis, IgG4-related lymphadenopathy, IgG4-related dacryoadenitis, IgG4-related sinonasal disease and IgG4-related hepatic mass. Kidney involvement was seen in three cases in combination with other organ manifestations [11, 13, 14]. Systemic IgG4-RD (two or more organ manifestations) occurred in 40 % of the cases [8, 11–19].

### Diagnosis

In this study, all cases of IgG4-RD were histologically confirmed, except one case of Riedel’s thyroiditis [20], whereby histology was performed without IgG4 staining. Riedel’s thyroiditis is recently included in the spectrum of IgG4-RD [21], therefore we decided to include this case report in this study. Furthermore, despite the presence of IgG4 positive plasma cells in the tissue, two case reports concerning Rosai-Dorfman disease and ALK-1 positive inflammatory myofibroblastic tumor [22, 23] were excluded, because according to Boston consensus these diseases should not be considered as IgG4-RD. Serum IgG4 was measured in 23 of the 25 cases, and was found to be elevated in 16 cases [5, 6, 8, 10, 12, 14–19, 24–27] (70 %).

### Therapy

Prednisone was the first choice of treatment in 23 of the 25 cases [5–11, 14–20, 24–29]. In one case no treatment was initiated or mentioned [12], and in another case surgery alone resulted in complete remission [7].

The doses of prednisone that was used were not mentioned in all cases, but when specified was usually between 0.5 and 2 mg/kg/day. Prednisone therapy resulted in a rapid response in 19 of the 23 cases treated [5–11, 13, 14, 17–20, 24–29]. Prednisone alone induced remission and could be tapered and discontinued without relapse in 10 of the cases (43 %), and thus was the sole agent used [6, 8, 18–20, 24, 26–29]. Second line therapy was initiated in the 4 cases (17 %) that did not respond completely to prednisone and in the 9 cases where prednisone alone did not induce permanent remission. In 3 of 4 cases not responding to prednisone, the prednisone doses were adequate, however, in 1 case the dosage was not mentioned. DMARDs were attempted as steroid-sparing agents in 11 cases. Mycophenolate mofetil was successful as a steroid-sparing agent in 3 of the 5 cases in which it was used [7, 9, 10, 14, 16]. Azathioprine was a successful as a steroid sparing agent in 2 of 4 cases in which it was used [5, 15, 17, 25], while methotrexate was successful in 1 of 2 cases [9]. Because of disease relapse despite azathioprine, one patient achieved clinical remission with 5 mg prednisone after high doses induction of prednisone [17].

Rituximab was initiated in 4 cases [8, 11, 13, 16] of therapy refractory diseases leading to positive clinical outcomes in all these cases. Two of these cases initiated rituximab single therapy [8, 13], in one case methylprednisolone was combined with rituximab [11] and in another case prednisone 10 mg daily was used as maintenance therapy beside rituximab [16]. Adalimumab [15] and cyclophosphamide [9] were both successfully used in therapy refractory cases.

## Discussion

In this systematic search of the literature we describe 25 published cases of IgG4-RD in children. The cases demonstrate different organ manifestations of the disease with different clinical outcomes emphasizing the broad clinical spectrum of this disease.

### Epidemiology

IgG4-RD is a rare and recently recognized fibro-inflammatory condition of which the diagnosis is often delayed or unrecognized because of unawareness. Generally it occurs in middle aged patients, more often in men than women [1]. However, in this study we identified more female patients than male patients. In children IgG4-RD is even more uncommon and will subsequently lead to significant delayed or unrecognized disease. All cases identified with this systematic review have been only recently published demonstrating that awareness is increasing in pediatricians. One can postulate that the average age of patients is lower than suggested [1], and may be more frequent in the pediatric age group than these 25 published cases might suggest.

### Symptoms and organ manifestation

The symptoms of IgG4-RD are variable and depend on the affected organs. It can be localized almost everywhere (Table 2). In adults, IgG4-RD mostly affects the orbit, the salivary tract, the pancreas and the lymph nodes, however, manifestations in almost every part of the human have been described [2]. In this study we have demonstrated a similar distribution of disease localizations in children. As in adults, most pediatric patients had orbital or pancreatic localizations. Therefore, IgG4-RD in children apparently is the same entity as in adults. In cases of unexplained inflammatory conditions, especially when tumor-like abnormalities are observed by physical examination or imaging studies in the preferential localization of the disease (pancreas, salivary glands, orbit, lymph nodes), one should rule out IgG4-RD. Furthermore, conditions previously called Mikulicz’ s disease, sclerosing sialadenitis, inflammatory orbital pseudotumor or any pseudotumor, a subset of idiopathic retroperitoneal fibrosis and Riedel’s thyroiditis are now mostly reclassified as IgG4-RD and should raise suspicion for IgG4-RD [30].

**Table 2:** Organ manifestations of IgG4-related disease

Pancreas Autoimmune pancreatitis type 1	Lymph nodes Ig4-related lymphadenopathy of several lymph nodes
Liver and bile duct IgG4-related sclerosing cholangitis IgG4-related cholecystitis IgG4-related hepatopathy	Other abdominal manifestations Inflammatory pseudotumors Retroperitoneal fibrosis Small bowel obstruction caused by peritoneal IgG4-RD IgG4-RD of stomach with chronic ulcer IgG4-related esophagitis
Kidneys Interstitial nephritis Glomerular lesions such as membranous nephropathy	Skin manifestation Erythematous, subcutaneous papules or nodules of IgG4 origin
Urological manifestation IgG4-related prostatitis Ureteral IgG4-RD Testicular inflammation as a manifestation of IgG4-RD	Orbital and ophthalmic manifestation Inflammatory pseudotumors of orbit Scleritis Retinopathy due to IgG4-RD with hypergammaglobulinemic hyperviscosity Trigeminal and orbital nerve compression Nasolacrimal duct obstruction
Pulmonary manifestation Interstitial lung disease/interstitial pneumonia Bronchial damage/asthma-like clinical presentation Plural manifestation of IgG4-disease Pulmonary arterial hypertension	Cardiovascular manifestation IgG4-related periaortitis IgG4-related aortitis Pericarditis IgG4-related coronary artery disease
Thyroid Riedel’s thyroiditis Fibrosing Hashimoto thyroiditis	Salivary and lacrimal gland IgG4-RD Mikulicz’s Küttner's tumor or IgG4-related submandibular gland disease
Nervous system Infundibular hypophyistis Hypertrophic pachymeningitis IgG4-related hypophysitis Intracerebral inflammatory pseudotumors Neuropathy	Other manifestations IgG4-related fibrosing mediastinitis IgG4-related myositis Multifocal fibrosclerosis Increased risk of malignancy: lung, colon and especially MALT lymphoma.

### Diagnosis

**Fig. 3:**
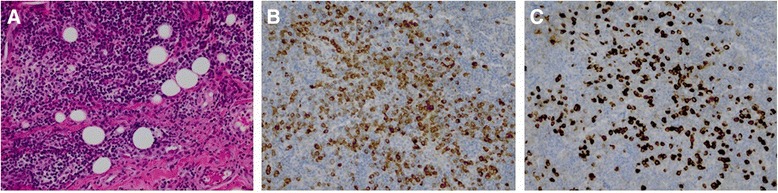
Histology of the orbital tissue of an adult patient from our hospital with IgG4-related orbital disease. **a** HE-staining demonstrating multiple lymphoid infiltrates and fibrosis. **b** Immunohistochemical staining for IgG showing diffuse scattered IgG (brown color). **c** Immunohistochemical staining for IgG4 revealing widely scattered IgG4 positive plasma cells (dark brown) with an average of 339 per HPF out of 2 HPF with a ratio of 0.67 to total IgG plasma cells in the tissue. HE = Hematoxylin and Eosin, HPF = High-power field

The diagnosis of IgG4-RD can only be confirmed histologically, the gold standard, while clinical symptoms, serological and radiological findings could be supportive to establish the diagnosis. The typical histological abnormalities (Fig. 3), according to the Boston consensus [31], are dense lymphoplasmacytic infiltrate, storiform fibrosis and obliterative phlebitis. The ratio of IgG4/IgG positive plasma cells in tissues should be greater than 0.4 and the numbers of IgG4 positive plasma cells per high power field (HPF) should be greater than the numbers agreed in the consensus [31]. IgG4 positive plasma cells in tissues could also be observed in several other conditions without meeting the histological diagnostic criteria for IgG4-RD. Therefore, alternative diagnosis such as xanthogranulomatous disease, granulomatosis with polyangiitis and sarcoidosis should be excluded before obtaining the diagnosis IgG4-RD [32]. In current study, almost all cases were histologically proven, except a case of Riedel’s thyroiditis, which is recently been recognized as a spectrum of IgG4-RD [21].

Serum IgG4 is elevated in most of the cases of IgG4-RD, but about 30 to 50 % of histologically confirmed cases have normal levels of serum IgG4, which can lead to falsely rejecting the diagnosis [30]. A similar percentage of pediatric patients had elevated serum IgG4 levels (70 %) to those reported in the adult population. In general the specificity and positive predictive value of serum IgG4 are low, but if elevated can be useful in monitoring response to treatment [31]. Inflammatory biomarkers such as erythrocyte sedimentation rate and C-reactive protein might be elevated, but normal levels of these biomarkers are frequently observed in IgG4-RD making them less specific as biomarkers [33]. Moreover, recently, serological studies of IgG4 positive circulating plasmablasts have been shown to be superior to serum IgG4 levels in IgG4-RD [34]. So far this technique has not been widely introduced for clinical applications.

### Pathogenesis

**Fig. 4:**
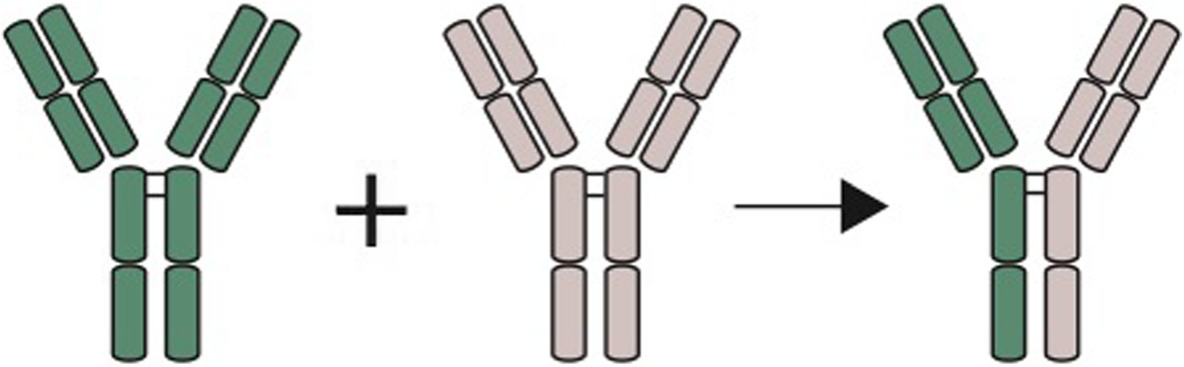
Proposed mechanism of the formation of IgG4 antibodies by ‘’Fab-Arm” exchange. IgG4 antibodies continuously exchange half molecules with other antibodies making them bivalent reactive antibodies with two different antigen-binding fragments. These antibodies are also unable to activate the classical complement system and can bind to antigens. However, as a result of bivalent reactivity unable to form immune complexes. Because of these characteristics the IgG4 antibodies are most probably anti-inflammatory agents rather than pro-inflammatory. Fab = antigen binding fragment

The pathogenesis of IgG4-RD is unclear. Generally abundant serological T-helper cells 2 and regulatory T-cells are observed. These are most probably induced by an antigen triggering the immune system [1]. Subsequently, interleukin (IL)-4,5,10,13 and transforming growth factor (TGF)-beta have been assumed to activate B-cells, hence producing IgG4 expressing B-cells and fibrosis [1]. The role of IgG4 antibodies in the pathogenesis is unclear, but because of characteristics of these antibodies [35], they most probably act as anti-inflammatory antibodies as response to an unknown trigger (Fig. 4).

### Treatment

When untreated, IgG4-RD can cause irreversible organ damage hence early and aggressive treatment is indicated [36]. Glucocorticoids are the first choice of the treatment for the adults, mostly effective at a prednisone dosage of 0.5 -1 mg/kg/day, adjusted according to aggressive disease [37]. In the presented study prednisone appeared first choice therapy for pediatric IgG4-RD. There is no consensus on prednisone dosage in pediatrics, but in general prednisone 1 to 2 mg/kg/day should be appropriate. Prednisone can thereafter be tapered according to individual response. Treatment with prednisone is often rapidly effective, but this treatment should be maintained for 2 to 4 weeks after initiation. In the presented study prednisone was generally effective first line therapy in 83 % of the cases. However, only in 43 % of the cases prednisone single therapy sufficed. The rest of the cases required maintenance (immunosuppressive) therapy. According to previous studies, especially on adults, about 25 % of patients show relapse of the disease despite prednisone maintenance therapy making steroid sparing agents necessary [37]. MMF, azathioprine and methotrexate were effective in about 50 % of the cases in this study. The role of DMARDs in the treatment of IgG4-RD is not yet clear and management of this disease with these agents has not been outlined [37]. Recently, increasing evidence for the efficacy of rituximab treatment of IgG4-RD has been demonstrated [38]. In this review four patients were treated with rituximab leading to significant clinical outcomes in all cases. We recommend rituximab as a strong alternative when a patient is refractory to therapy. Intravenous or subcutaneous immunoglobulin treatment has been successfully used in other inflammatory or immune mediated diseases, but this therapy has not yet been applied in IgG-RD [39].

Serum IgG4, when elevated, can be used in disease activity monitoring after initiating treatment, however, the role of serum IgG4 as disease activity marker has not yet fully been outlined [1]. Studies should define the role of serum IgG4 as disease marker, same applies to circulating plasmablasts. Imaging studies, especially PET scan is useful in disease monitoring. Studies have shown the usefulness of FDG-PET/CT scan for diagnosis, staging and the degree of organ involvement and monitoring of therapy response, and this imaging method seems to detect more lesions than conventional methods like ultrasonography and CT [40].

## Conclusion

In conclusion, IgG4-RD is a relatively new disease and generally unknown to pediatricians. The results of this study suggest that the average age of patients is lower than suggested in the literature. Early recognition and therapy are important to prevent serious and irreversible organ damage. Treatment with prednisone is the first choice for this disease, but maintenance therapy with DMARDs is often required. Rituximab may be a good alternative in therapy refractory disease. Further (epidemiological) studies should confirm these preliminary conclusions. Moreover, serological and histological studies and studies on treatment of children with IgG4-RD are needed in order to confirm the same results in children compared with previous studies performed in adults.

## Appendix

Search terms used in the medical databases for the literature search in this systematic review on IgG4-RD in pediatrics.

### Embase.com (251)

(‘immunoglobulin G4 related disease’/exp OR ‘Mikulicz disease’/exp OR ((G4 OR igg4 OR ‘igg 4′ OR Mikulicz OR kuttner OR riedel*) NEAR/3 (rd OR related OR associat* OR autoimmun* OR disease* OR inflammat* OR tumor* OR thyroidit*)):ab,ti) AND (child/exp OR newborn/exp OR adolescent/exp OR adolescence/exp OR ‘child behavior’/de OR ‘child parent relation’/de OR pediatrics/exp OR childhood/exp OR ‘child nutrition’/de OR ‘infant nutrition’/exp OR ‘child welfare’/de OR ‘child abuse’/de OR ‘child advocacy’/de OR ‘child development’/de OR ‘child growth’/de OR ‘child health’/de OR ‘child health care’/exp OR ‘child care’/exp OR ‘childhood disease’/exp OR ‘child death’/de OR ‘child psychiatry’/de OR ‘child psychology’/de OR ‘pediatric ward’/de OR ‘pediatric hospital’/de OR ‘pediatric nursing’/exp OR ‘pediatric anesthesia’/exp OR ‘pediatric surgery’/exp OR (adolescen* OR infan* OR newborn* OR (new NEXT/1 born*) OR baby OR babies OR neonat* OR child* OR kid OR kids OR toddler* OR teen* OR boy* OR girl* OR minors OR underag* OR (under NEXT/1 (age* OR aging)) OR juvenil* OR youth* OR kindergar* OR puber* OR pubescen* OR prepubescen* OR prepubert* OR pediatric* OR paediatric* OR school* OR preschool* OR highschool* OR picu OR nicu OR picus OR nicus):ab,ti)

### Medline (224)

(Mikulicz’ Disease/OR ((G4 OR igg4 OR igg 4 OR Mikulicz OR kuttner OR riedel*) ADJ3 (rd OR related OR associat* OR autoimmun* OR disease* OR inflammat* OR tumor* OR thyroidit*)).ab,ti.) AND (exp child/OR exp infant/OR adolescent/OR exp pediatrics/OR exp Child Health Services/OR Hospitals, Pediatric/OR (adolescen* OR infan* OR newborn* OR (new ADJ born*) OR baby OR babies OR neonat* OR child* OR kid OR kids OR toddler* OR teen* OR boy* OR girl* OR minors OR underag* OR (under ADJ (age* OR aging)) OR juvenil* OR youth* OR kindergar* OR puber* OR pubescen* OR prepubescen* OR prepubert* OR pediatric* OR paediatric* OR school* OR preschool* OR highschool*).ab,ti.)

### Cochrane (6)

(((G4 OR igg4 OR ‘igg 4’ OR Mikulicz OR kuttner OR riedel*) NEAR/3 (rd OR related OR associat* OR autoimmun* OR disease* OR inflammat* OR tumor* OR thyroidit*)):ab,ti) AND ((adolescen* OR infan* OR newborn* OR (new NEXT/1 born*) OR baby OR babies OR neonat* OR child* OR kid OR kids OR toddler* OR teen* OR boy* OR girl* OR minors OR underag* OR (under NEXT/1 (age* OR aging)) OR juvenil* OR youth* OR kindergar* OR puber* OR pubescen* OR prepubescen* OR prepubert* OR pediatric* OR paediatric* OR school* OR preschool* OR highschool* OR picu OR nicu OR picus OR nicus):ab,ti)

### Web-of-science (126)

TS = ((((G4 OR igg4 OR “igg 4” OR Mikulicz OR kuttner OR riedel*) NEAR/2 (rd OR related OR associat* OR autoimmun* OR disease* OR inflammat* OR tumor* OR thyroidit*))) AND ((adolescen* OR infan* OR newborn* OR (new NEAR/1 born*) OR baby OR babies OR neonat* OR child* OR kid OR kids OR toddler* OR teen* OR boy* OR girl* OR minors OR underag* OR (under NEAR/1 (age* OR aging)) OR juvenil* OR youth* OR kindergar* OR puber* OR pubescen* OR prepubescen* OR prepubert* OR pediatric* OR paediatric* OR school* OR preschool* OR highschool* OR picu OR nicu OR picus OR nicus)))

### PubMed publisher (33)

(Mikulicz’ Disease[mh] OR ((G4[tiab] OR igg4[tiab] OR “igg 4”[tiab] OR Mikulicz[tiab] OR kuttner[tiab] OR riedel*[tiab]) AND (related[tiab] OR associat*[tiab] OR autoimmun*[tiab] OR disease*[tiab] OR inflammat*[tiab] OR tumor*[tiab] OR thyroidit*[tiab]))) AND (child[mh] OR infant[mh] OR adolescent[mh] OR pediatrics[mh] OR Child Health Services[mh] OR Hospitals, Pediatric[mh] OR (adolescen*[tiab] OR infan*[tiab] OR newborn*[tiab] OR new born*[tiab] OR baby OR babies OR neonat*[tiab] OR child*[tiab] OR kid OR kids OR toddler*[tiab] OR teen*[tiab] OR boy*[tiab] OR girl*[tiab] OR minors OR underag*[tiab] OR under age*[tiab] OR under aging*[tiab] OR juvenil*[tiab] OR youth*[tiab] OR kindergar*[tiab] OR puber*[tiab] OR pubescen*[tiab] OR prepubescen*[tiab] OR prepubert*[tiab] OR pediatric*[tiab] OR paediatric*[tiab] OR school*[tiab] OR preschool*[tiab] OR highschool*[tiab])) AND (publisher[sb] OR inprocess [sb])

### Google scholar (100)

“G4|igg4|Mikulicz|kuttner|riedel rd|related|associat|autoimmune|disease|inflammation|tumor|thyroiditis” child|children|adolescent|adolescents|adolescence|infant|infants|infancy

## Abbreviations

DMARDs: disease modifying antirheumatic drugs
Fab: antigen binding fragment
HE: hematoxylin and Eosin
HPF: high-power field
IgG4-RD: immunoglobulin G4-related disease
